# Clinicopathologic Significance of CXCL12 and CXCR4 Expressions in Patients with Colorectal Cancer

**DOI:** 10.1155/2018/9613185

**Published:** 2018-05-16

**Authors:** Naomi Yoshuantari, Didik Setyo Heriyanto, Susanna Hilda Hutajulu, Johan Kurnianda, Ahmad Ghozali

**Affiliations:** ^1^Department of Anatomical Pathology, Faculty of Medicine, Public Health, and Nursing, Universitas Gadjah Mada/Dr. Sardjito General Hospital, Yogyakarta, Indonesia; ^2^Division of Hematology and Medical Oncology, Department of Internal Medicine, Faculty of Medicine, Public Health, and Nursing, Universitas Gadjah Mada/Dr. Sardjito General Hospital, Yogyakarta, Indonesia

## Abstract

**Background:**

Colorectal cancer (CRC) is both a global and national burden, being the third most common malignancy in men and the second in women, worldwide. The prognosis of CRC is affected by various factors like the histological grade, angiolymphatic invasion, and distant metastases. Metastasis is an intricate process; one of the possible mechanisms is through the interaction of the chemokines CXCL12 and CXCR4. This study aims to reveal the expression patterns of CXCL12 and CXCR4 in CRC.

**Methods:**

The quantitative expressions of CXCL12 and CXCR4 messenger RNA (mRNA) were evaluated in 32 patients with adenocarcinoma-type CRC. Real-time polymerase chain reaction (qRT-PCR) was performed on formalin-fixed tissues. CXCL12 and CXCR4's expressions, clinicopathologic features, and the treatment response to the CRC were analysed.

**Results:**

All tumour tissues showed higher levels of both chemokines compared to normal colonic tissue. The expression of CXCL12 mRNA was higher in rectal location (*p* = 0.04) with a tendency to be higher in later stages (*p* = 0.15), while the expression of CXCR4 was lower in tumours with a lymphatic invasion (*p* = 0.02), compared to their counterparts. There was no difference in the expression of CXCL12 and CXCR4 according to the patients' ages, gender, tumour differentiation, or response to chemotherapy.

**Conclusion:**

Our study demonstrated that the mRNA expression of CXCL12 was significantly correlated with rectal location. CXCR4 mRNA expression was inversely correlated in tumours with a lymphatic invasion.

## 1. Introduction

Colorectal cancer (CRC) is the third most prevalent malignancy among men and the second among women, worldwide. In Indonesia, CRC is the second most frequent cancer in men after lung cancer and the third in women, after breast and cervical cancer [1]. The dominant histological type of this malignancy is adenocarcinoma. Cancer-related mortality is generally caused by local recurrences and metastasis [2]. Patients with CRC have a good prognosis when they are diagnosed early, before metastatic lesions develop [1, 3]. However, only 40% of cases are found at the early stages [2]. Despite new chemotherapeutic regimens, CRC continues to present a progressive outcome [4]. Early metastatic pathological signs include vascular emboli, lymphatic invasion, perineural invasion, or multiple presentations [5]. During the process of metastasis, tumour cells detach from their primary nest, enter the angiolymphatic systems and other organs, and then adhere to the endothelial cells; one of these mechanisms is by adhering to chemokine receptors [6].

Chemokine, a chemotactic cytokine which mediates leucocyte migration (chemotaxis), is a small-sized protein expressed by various cells (leucocytes, epithelial cells, endothelial cells, and fibroblasts), including tumour cells [3, 7]. Chemokine is classified into 4 groups according to its terminal residual cysteine position: CXC, CC, C, and CX_3_C. Of all groups, CXC plays a very important role in angiogenesis [5]. The CXC chemokine is further sorted based on its ELR pattern (Glu, Leu, and Arg), namely ELR^+^ and ELR^−^. ELR^+^ is angiogenic and chemotactic against neutrophils. ELR^−^ has an inhibitory effect towards angiogenesis and attracts lymphocytes and natural killer cells [8, 9]. ELR^−^ chemokines are commonly angiostatic, but CXCL12 (previously known as stromal cell-derived factor 1, abbreviated as SDF1) [10] and its receptor, CXCR4 (previously known as LESTR, fusion, or CD184), are reported to promote angiogenesis and play a major role in metastasis.

The interaction of CXCL12 and CXCR4 has been addressed as engaging in the tumour progression of various cancers [8, 10], including CRC [11]. The CXCL12 and CXCR4 axis plays a role in the metastatic homing of tumour cells. A high CXCR4 expression can promote lymph node metastasis by the migration mechanism, in cooperation with CXCL12 [12]. Multiple studies show a link between the CXCL12/CXCR4 pathway and CRC [8, 13–20]. However, there are contradictive reports on the expression level of CXCL12 and CXCR4 mRNA in CRC, especially when compared to normal colonic mucosa. Some studies observed a decrease in the expression level, while others addressed the marked increase in the expression of both chemokines in CRC [21]. In addition, other reports have observed that the expression of CXCR4 is associated with the clinical stage, lymph node metastasis, and liver metastasis which could assist in determining the prognosis [22, 23]. To date, reports on CXCL12 and CXCR4 expression in the Indonesian population have only come from studies of patients with breast cancer [24], but not yet from patients with CRC. Therefore, the present study aims to explore the CXCL12 and CXCR4 expression profile in local CRC patients and determine their association with various clinicopathologic factors such as age, tumour differentiation, angiolymphatic and perineural invasion, and their response to chemotherapy.

## 2. Methods

### 2.1. Clinical, Pathological, Treatment, and Evaluation Data

This study recruited data of 32 eligible patients who were diagnosed with colorectal adenocarcinoma between 2006 and 2015. Surgical specimens and corresponding normal tissue samples were collected from the archive of the Anatomical Pathology Laboratory. The study was approved by the Institutional Review Board (IRB) of Universitas Gadjah Mada/Dr. Sardjito General Hospital (reference number KE/FK/982/EC/2016).

Data of the clinicopathological characteristics, treatment, and response evaluation were extracted from the medical records. Clinical variables include the sex (male versus female), age (using a median point of 55 years, with ≤55 being defined as younger versus >55 years defined as older), and the clinical tumour nodal metastasis (TNM) stage. Since there was no stage I in this local panel, the patients were furthered classified as stage II, stage III, and stage IV [18]. Pathological variables include tumour differentiation (well, moderate, and poor), angioinvasion (yes versus no), and perineural invasion (yes versus no).

The treatment setting for the local patients with CRC was performed as either an adjuvant or a metastatic scheme. Adjuvant chemotherapy included 5-fluoropyrimidin-based regimens such as 5-FU combined with leucovorin (5-FU/LV), capecitabine, or 5-FU/LV combined with oxaliplatin (FOLFOX or XELOX) [25]. Metastatic chemotherapy included bevacizumab-based or nonbevacizumab-based regimens [26]. The response's evaluation was divided into four groups based on the World Health Organization (WHO) tumour response criteria [27]. A complete response was defined as when there were no clinical symptoms or no tumours visible on the sigmoidoscopy or colonoscopy evaluation. A partial response was defined as shrinkage of the tumour to less than 50% of its previous size, without clinical symptoms. A stable disease was defined as there being neither a partial response nor the progressive disease criteria being met. The progressive disease status was for cases with locally advanced and metastatic patients [27]. Treatment evaluation was further classified as a good outcome for cases with a complete response or a partial response and a poor outcome for cases with a stable response or the progressive disease classification. For patients treated under the metastatic scheme, this evaluation was done regardless of the treatment in the adjuvant setting [28].

### 2.2. Tissue Preparation

Tissue specimens were collected from formalin-fixed paraffin-embedded tissues of resection surgical procedures. Tumour samples were taken from vital areas of histopathologically confirmed CRC. For normal tissue, we utilised adjacent unaffected mucosa, generally distal to the resection margin. All tissues were reviewed for the presence of tumour cells. The remaining normal tissues were scraped off the slides.

### 2.3. Real-Time Polymerase Chain Reaction (RT-PCR)

All the quantitative real-time PCR assays containing the primer and probe mix were purchased from Gene-All Hybrid and utilised according to the manufacturer's instructions. Real-time PCRs were done using One-Step qRT-PCT with KAPA SYBR FAST Universal. Reactions of the PCRs were carried out using the forward primer GAPDH (5′-GCA TCC TGG GCT ACA CTG AG-3′), CXCL12 (5′-GAT TGT AGC CCG GCT GAA GA-3), and CXCR4 (5′-AGC ATG ACG GAC AAG TAC C-3′) and the reverse primer GAPDH (5′TCC ACC CTG TTG CTG TA-3′), CXCL12 (5′ TTC GGG TCA ATG CAC ACT TGT-3), and CXCR4 (5′GAT GAT ATG GAC ACC CTT ACA C-3′). An individual reaction was performed using the DT-Lite Real-Time PCR System (DNA Technology) with reverse transcription at 42°C for 5 minutes, followed by enzymatic activation at 95°C for 3 minutes, denaturation for 1–3 seconds at 95°C, and elongation for up to 20 seconds at 60°C.

The normal tissue became the 1x sample, and all the other quantities were expressed as the *n*-fold difference relative to this tissue as the control. The expression rates of CXCL12 and CXCR4 were counted using the formula: 2^(−*ΔΔ*CT)^, with *Δ*CT = PCR score of CXCL12 or CXCR4-PCR score GAPDH. ΔΔCT = ΔCT of tumour sample − ΔCT normal colon tissue [29].

### 2.4. Statistical Analysis

The data were analysed using SPSS for Macintosh OS × 10.12 (Standard GradPack version 24; SPSS Inc., Chicago, Il, USA). Due to CXCL12's and CXCR4's distribution, a logarithmic transformation was performed. The association between the mRNA expression of CXCL12 and CXCR4 and the clinicopathologic factors were assessed using an independent *t*-test for normally distributed data and the Mann–Whitney *U* test for nonnormally distributed data. The strength of the link was then assessed using Pearson's correlation. Normally distributed multivariate factors were assessed using a one-way ANOVA and nonparametric (2-independent and *K*-independent) tests for nonnormally distributed data. *p* values < 0.05 were considered significant.

## 3. Results

### 3.1. Patient Characteristics

Formalin-fixed paraffin-embedded specimens from the tumours of 32 patients resected for CRC were studied. The clinicopathologic features of the patients and the treatment responses are summarised in Table 1. Twenty patients were male (62.5%) and 12 were female (37.5%). The median age at diagnosis was 55 years (range 30 to 66 years), 16 patients (50%) were ≤55 years old, and 16 patients (50%) were >55 years old. Based on tumour locations, there were 26 patients with tumours located in the colon (81.3%), whereas only 6 patients with tumours located in the rectum (18.8%). According to the TNM clinical stage, 17 patients were in stage II (53.2%), 10 patients in stage III (31.3%), and 5 patients in stage IV (15.6%). There were 2 patients who had liver metastases, and 1 patient with lung metastasis. According to the tumour's differentiation, there were 12 patients with a well differentiation (37.5%), 17 patients with a moderate differentiation (53.1%), and 3 patients with a poor differentiation (9.4%). All recruited tumours were adenocarcinoma, and 3 of them were with mucoid features (9.4%). In the adjuvant setting, most of the patients were treated with oxaliplatin-based chemotherapy (29 patients, 90.63%) while 2 patients (6.25%) were administered with nonoxaliplatin-based regimens and 1 patient with missing data. For all oxaliplatin-based chemotherapy, the XELOX regimen was administered to 28 patients (87.5%), and FOLFOX regimen was given to one patient (3.1%). One patient was treated with 5-FU/LV only, and one patient received capecitabine only.

### 3.2. CRC Cells Expressed as CXCL12 and CXCR4

**Figure 1:**
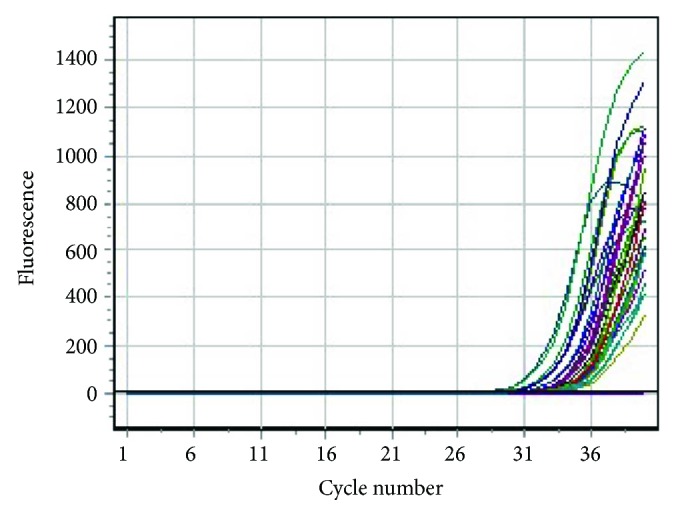
Dependence of FAM channel fluorescence on cycle number. Real-time PCR assay result showed the expression of the CXCL12 and CXCR4 mRNA. Each line represents the expression of assay wells which occurred approximately around cycle number 31, at different levels. The strongest expressions were on the left-most (earliest) part of the curves.

The results of the quantitative RT-PCR analysis are shown in Figure 1. We utilised normal colonic tissue as a control for the CXCL12 and CXCR4's expression levels and compared the expressions with the CRC tissue. We noticed a wide range of expression levels for both chemokines. The mean level of the CXCL12's expression was 12.46 (range 2–56), and the mean level of the CXCR4's expression was 8.24 (range 1–49). Compared to normal colonic tissue that is considered to have a 1-fold expression level, most of the tumour tissues had increased levels. This wide range might be caused by some factors; one probable cause was because this study performed a total RNA extraction from the archival paraffin-embedded formalin-fixed tissues. RNA is extensively degraded by routine formalin fixation into fragments averaging 200 nucleotides [30]. Another probable cause would be the different lengths of storage of the samples; those acquired from 2006 were probably inappropriately stored. Besides, some samples were obtained from laboratories outside of the Dr. Sardjito General Hospital that might have applied different methods of storage.

### 3.3. Correlation of CXCL12 and CXCR4 Levels with Clinicopathologic Parameters

**Figure 2:**
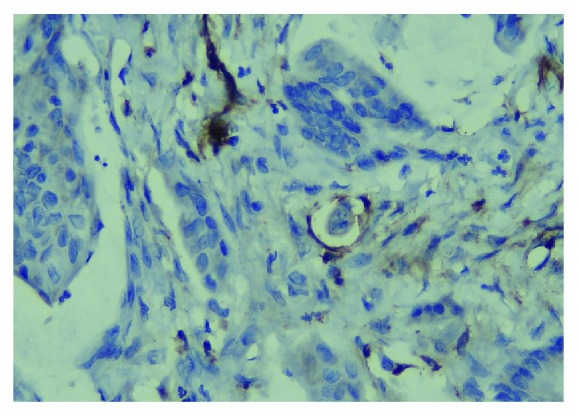
Lymphatic invasion shown by the presence of tumour cells inside the lymphatic vessel stained by D2-40 immunohistochemical staining.

**Figure 3:**
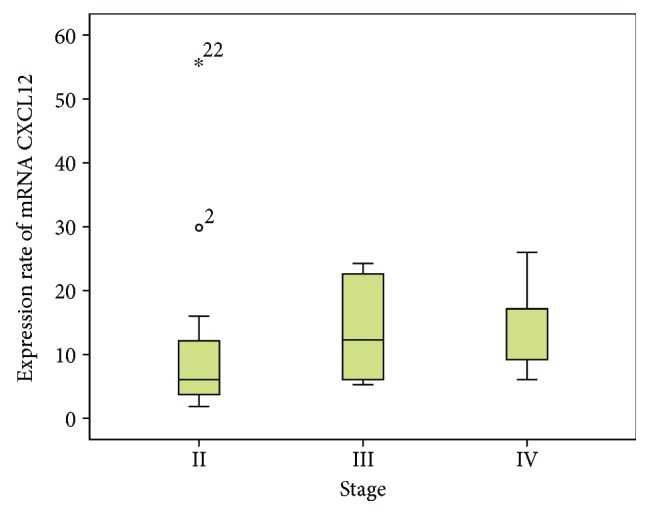
The level of the CXCL12 mRNA gene expression showed a tendency to become higher in later stages compared to the earlier stages (*p* = 0.15).

**Figure 4:**
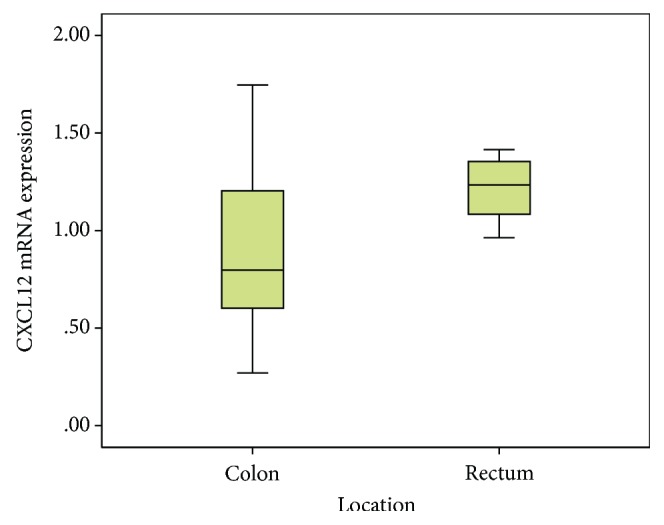
The level of the CXCL12 mRNA gene expression was significantly higher in rectal location compared to colon location (*p* = 0.04).

**Figure 5:**
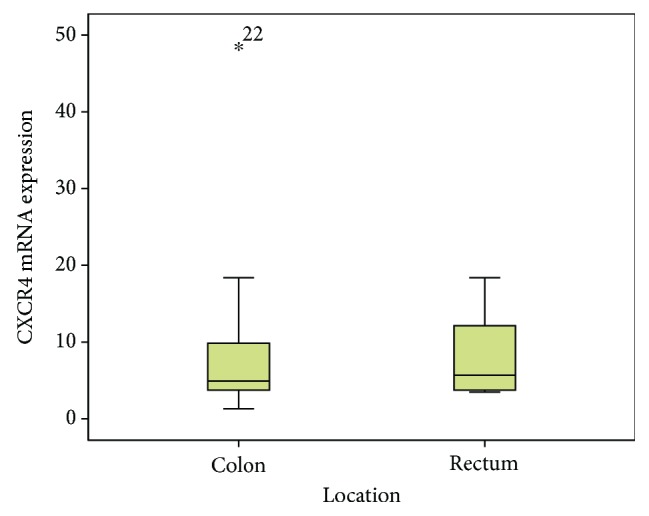
The level of the CXCR4 mRNA gene expression was slightly higher in rectal location compared to colon location (*p* = 0.71).

**Figure 6:**
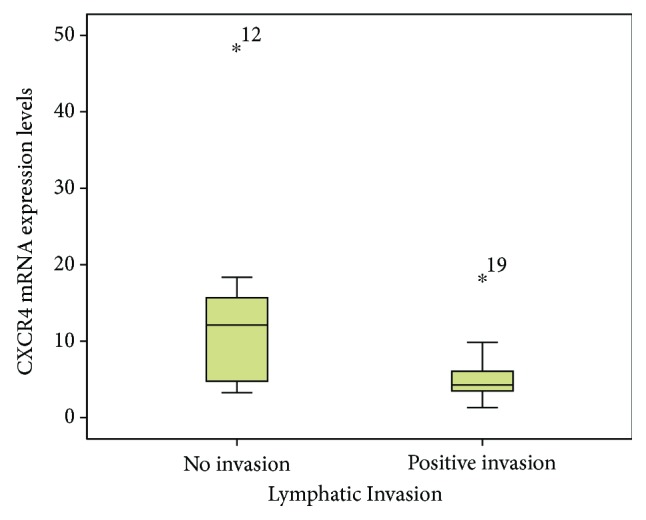
The level of the CXCR4 mRNA gene expression was significantly lower in tumours with a positive intratumoural lymphatic invasion compared to tumours without an intratumoural lymphatic invasion (*p* = 0.02).

Table 2 displays the CXCL12 and CXCR4 levels across the clinicopathologic parameters and treatment outcomes.  Figure 2 shows the presence of tumour cells inside the lymphatic channel, emphasised by immunohistochemical staining using D2-40 (podoplanin). The expressions of CXCL12 and CXCR4 were higher in women and younger patients, in stage IV and stage III cases (late stage tumours), in tumours located in the rectum, in tumours with moderate and poor histology differentiation, and tumours without angioinvasion, lymph invasion, and perineural invasion, compared to their counterparts. For all the clinical and pathological parameters, the expressions of both chemokines were consistent. A higher level of CXCL12 was shown in cases with stage IV, followed by stage III, and the least in stage II (*p* = 0.15, Figure 3). Tumours located in the rectum had a higher expression of CXCL12 compared to tumours located in the colon (Figure 4). The CXCR4 expression in patients with lymphatic invasion was slightly higher in rectal tumour locations than in the colon (*p* = 0.71) as shown in Figure 5. Expression of CXCR4 was significantly lower compared with that of tumours without invasion (*p* = 0.02, Figure 6). A significant moderate, inverse linear negative correlation was observed between expression of the level of CXCR4 with lymphatic invasion (*p* = 0.02, *r* = −0.44). There was also a tendency of a lower expression of CXCR4 in tumours with angioinvasion (*p* = 0.07) with a moderate negative linear correlation (*r* = −0.37, *p* = 0.07). When analyses of the angioinvasion and lymph invasion were merged into an angiolymphatic invasion, the negative linear correlation became stronger (*r* = −0.49, *p* = 0.013).

## 4. Discussion

Chemokines act as inflammatory mediators and function in the cross-communication of tumour cells. Studies have suggested that chemokines are involved in the pathogenesis of metastasis in numerous cancers [10]. In the chemokine superfamily, there is a multiple connection between ligands and numerous receptors, except in CXCL12 and CXCR4, which only has one pair and does not cross with other ligands or receptors [21, 31]. Recent studies demonstrated that the CXCL12-CXCR4 axis has been linked to diverse human tumour types, including breast cancer [32, 33], ovarian cancer [34], melanoma [35], gastric cancer [36], non-small-cell lung cancer [37], multiple myeloma [38], and colorectal cancer. Our study demonstrates that both the chemokine ligand CXCL12 and its receptor CXCR4 are expressed by colorectal tumour cells.

It has been proposed that tumour cells will metastasise to locations where they are chemoattracted and arrested by locally secreted chemoattractants. Following appropriate microenvironmental conditions, tumour cells will survive and be able to proliferate. The microenvironment of tumours has been a recent focus of various cancer researches [10, 37, 39]. The progression of a tumour during carcinogenesis can be related to its intratumoural hypoxia by upregulating the CXCR4 expression in the tumour cells. This mechanism keeps the CXCR4 receptor protein levels high. It is reported that CXCR4 is strongly expressed in hypoxic conditions by the regulation of HIF-1*α*. High levels of CXCL12 in tumour tissue will invite CXCR4-positive inflammatory, endothelial, and stromal cells into the tumour. This will support the growth of tumour mass by inducing growth factors, cytokines, and proangiogenic factors assisting tumour invasion. In our study, although there were no significant differences across stages, there was a tendency of higher CXCL12 expressions which correlated with increasing stage [10, 37, 40].

It has been postulated that CXCR4 has a maintenance role in renewing colonic epithelium. Colorectal tumour cells' differentiation induced the downregulation of CXCR4. CXCL12 production in the early stage of CRC may assist angiogenesis and the growth of tumour cells, while in subsequent stages, CXCL12 production is lower, in order to avoid the recruitment of cytotoxic lymphocytes and enhancing the metastatic potential of the CRC tumour cells towards sites producing high levels of CXCL12 [19]. CXCL12 also assists the survival or growth of normal or malignant cells [37].

Verbeke et al. observed that CXCL12 encourages the metastasis of CXCR4-positive tumour cells to distant sites by angiogenesis. However, the influence of communications between normal stromal cells and CRC tumour cells may affect the expression pattern of both cell types [8]. CXCL12 is expressed by normal colon epithelial cells; thus, it is possible that this ligand aids the spreading of tumour cells to the normal colon epithelium [19]. CXCL12 and CXCR4 have been identified with significantly elevated levels in various malignancies and correlate with the survival, proliferation, angiogenesis, and the metastasis of tumour cells. Thus, studies suggest the inhibition of the CXCL12/CXCR4 axis as a targeted therapy [36]. Our study showed a similar result which is a tendency of higher CXCL12 levels in tumours at late clinical stages and significant higher CXCL12 levels in the rectum. However, lower CXCR4 levels were observed in more advanced tumours.

Recent studies have shown that CXCR4 has different expression patterns of tumour cells, which suggests a different biological behaviour by cancer. CXCR4 is a cell surface receptor yet various studies show that it can be expressed at different sites with different tumour behaviour [41, 42]. The expression of CXCR4 in the nuclear, cytoplasmic, and the membrane staining of tumour cells all have a different prognosis. A high cytoplasmic expression in the cytoplasm or membrane of the tumour cells indicates a worse prognosis, whereas a high nuclear expression indicates a better prognosis [13, 43]. However, our study did not explore the immunohistochemical staining profiles of CXCR4 or CXCL12.

Colorectal cancer cases are first diagnosed at an advanced stage in most cases, with some even showing metastasis. CXCR4 expression is closely associated with lymph node metastasis, the clinical stage, and the histological grade in CRC and other gastrointestinal cancers such as gastric cancer [22, 36, 44, 45]. Furthermore, a study by Kim and colleagues described that a high expression of CXCR4 in CRC tissues was linked to local recurrences, whereas tissues with low CXCR4 expressions had none [23]. Our present study found the association of CXCR4 expressions with a lymphatic invasion, but not with the clinical stage, the histological grade, or any recurrences.

Multiple studies reported a contradictory association between the patterns of CXCL12 expression levels with the patients' survival. While several studies reported that a higher CXCL12 expression in CRC is associated with a higher tumour stage, the prevalence of lymphatic invasion, venous invasion, lymph node metastasis [46, 47], and others reported that a high or strong expression of CXCL12 was associated with a better 5-year-disease-free survival [48]. Although we did not analyse the patients' survival, our findings showed that CXCL12 was highly expressed in patients with a worse clinical outcome. This was also supported by its higher expression in patients in stage IV of the disease. Our study also showed significant higher expressions in rectal locations [49]. We did not observe any significant correlation of CXCL12's expression with lymphatic and angioinvasion. It is also compelling that CXCL12 and CXCR4 were both expressed more highly in the tumours of patients without angioinvasion or lymphatic invasion and vice versa.

Here, we provided a report for the clinical use of CXCR4 expressions in CRC. Our study can provide preliminary data of CXCR4 and CXCL12 expressions of CRC in Asians, especially the Indonesian population. Albeit limited by the small size of the sample, our study showed fascinating results, which differ from previous studies. A larger sample is needed for any subsequent research. Finally, more extensive studies of chemokines in CRC patients, their ligands, and receptors, especially in Indonesia, are required to untangle the intricate interrelation of chemokines in tumours' development and progression. This in turn may reveal a clinical therapeutic application in CRC patients.

## 5. Conclusion

In summary, the present study showed that the expression of CXCL12 mRNA is higher in rectal tumours and CXCR4 mRNA was inversely correlated in colorectal tumours with a lymphatic invasion.

## Figures and Tables

**Table 1:** Clinicopathological and treatment characteristics of patients with CRC.

Variables	Frequency (%)
Gender	
Male	20 (62.5)
Female	12 (37.5)
Age	
Younger (≤55 years)	16 (50)
Older (>55 years)	16 (50)
Clinical stage	
II	17 (53.2)
III	10 (31.3)
IV	5 (15.6)
Location	
Colon	26 (81.3)
Rectum	6 (18.8)
Tumour differentiation	
Well	12 (37.5)
Moderate	17 (53.2)
Poor	3 (9.3)
Histological type	
Adenocarcinoma	29 (90.6)
Adenocarcinoma with mucinous features	3 (9.3)
Chemotherapy in adjuvant setting	
Oxaliplatin-based	29 (90.6)
Nonoxaliplatin-based	2 (6.3)
Missing data	1 (3.1)
Treatment response	
Complete remission	17 (53.2)
Partial remission	0 (0)
Stable disease	1 (3.1)
Progressive disease	14 (43.7)

**Table 2:** Association of expression level of CXCL12 and CXCR4 and clinicopathological characters.

Factor	CXCL12 level	*p* value	CXCR4 level	*p* value
Sex				
Men	7.48	0.12	5.76	0.62
Women	12.05		6.60	
Age				
Younger (≤55 years)	10.29	0.32	6.20	0.86
Older (>55 years)	7.63		5.91	
Clinical stage				
II	6.87	0.15	5.90	0.83
III	11.46		6.76	
IV	13.35		5.34	
Locations				
Colon	7.78	**0.04**	5.93	0.71
Rectum	16.36		6.7	
Tumour differentiation				
Well differentiation	7.58	0.69	17.50	0.43
Moderate differentiation	9.96		16.97	
Poor differentiation	9.39		9.83	
Angioinvasion				
No invasion	11.97	0.24	7.04	0.07
With invasion	7.60		3.72	
Lymphatic invasion				
No invasion	14.06	0.28	6.27	**0.02**
With invasion	9.29		4.74	
Perineural invasion				
No invasion	11.04	0.61	6.27	0.47
With invasion	8.97		4.74	
Clinical outcome				
Good	8.60	0.78	7.01	0.24
Poor	9.35		5.15	

## Data Availability

The data that support the findings of this study are available on request from the corresponding author (Ahmad Ghozali). The data are not publicly available due to the aforementioned data containing information that could compromise research participant privacy/consent.
